# A Complex Interplay Between Melatonin and RORβ: RORβ is Unlikely a Putative Receptor for Melatonin as Revealed by Biophysical Assays

**DOI:** 10.1007/s12035-024-04395-y

**Published:** 2024-08-06

**Authors:** Jiraporn Panmanee, Sitthivut Charoensutthivarakul, Chew Weng Cheng, Kornkanok Promthep, Sujira Mukda, Tanya Prasertporn, Chutikorn Nopparat, Kittitat Teerapo, Promsup Supcharoen, Nopphon Petchyam, Banthit Chetsawang, Piyarat Govitrapong, Matthew Phanchana

**Affiliations:** 1 Research Center for Neuroscience, Institute of Molecular Biosciences, Mahidol University, Nakhon Pathom, 73170 Thailand; 2 Innovative Molecular Discovery Laboratory (iMOD), School of Bioinnovation and Bio-Based Product Intelligence, Faculty of Science, Mahidol University, Bangkok, 10400 Thailand; 3 Excellent Center for Drug Discovery (ECDD), Faculty of Science, Mahidol University, Bangkok, 10400 Thailand; 4 Center for Neuroscience, Faculty of Science, Mahidol University, Bangkok, 10400 Thailand; 5 Leeds Institute of Cardiovascular and Metabolic Medicine, Faculty of Medicine and Health, University of Leeds, Leeds, LS2 9JT UK; 6 Innovative Learning Center, Srinakharinwirot University, Sukhumvit 23, Bangkok, 10110 Thailand; 7 Mahidol University-Frontier Research Facility (MU-FRF), Mahidol University, Nakhon Pathom, 73170 Thailand; 8 Center for Advanced Therapeutics, Institute of Molecular Biosciences, Mahidol University, Nakhon Pathom, 73170 Thailand; 9 Chulabhorn Graduate Institute, Kamphaeng Phet 6 Road, Lak Si, Bangkok, 10210 Thailand; 10 Department of Molecular Tropical Medicine and Genetics, Faculty of Tropical Medicine, Mahidol University, Bangkok, 10400 Thailand

**Keywords:** Melatonin, Nuclear Receptor, RORβ, Melatonin Receptors, Pineal Gland

## Abstract

A nuclear retinoic acid receptor (RAR)-related orphan receptor β (RORβ) is strictly expressed in the brain, particularly in the pineal gland where melatonin is primarily synthesized and concentrated. The controversial issues regarding the direct interaction of melatonin toward ROR receptors have prompted us to investigate the potential melatonin binding sites on different ROR isoforms. We adopted computational and biophysical approaches to investigate the potential of melatonin as the ligand for RORs, in particular RORβ. Herein, possible melatonin binding sites were predicted by molecular docking on human RORs. The results showed that melatonin might be able to bind within the ligand-binding domain (LBD) of all RORs, despite their difference in sequence homology. The predicted melatonin binding scores were comparable to binding energies with respect to those of melatonin interaction to the well-characterized membrane receptors, MT1 and MT2. Although the computational analyses suggested the binding potential of melatonin to the LBD of RORβ, biophysical validation failed to confirm the binding. Melatonin was unable to alter the stability of human RORβ as shown by the unaltered melting temperatures upon melatonin administration in differential scanning fluorometry (DSF). A thermodynamic isothermal titration calorimetry (ITC) profile showed that melatonin did not interact with human RORβ in solutions, even in the presence of SRC-1 co-activator peptide. Although the direct interaction between the LBD of RORβ could not be established, RORα and RORβ gene expressions were increased upon 24 h treatment with μM-range melatonin. Our data, thus, support the studies that the nuclear effects of melatonin may not be directly mediated via its interaction with the RORβ. These findings warrant further investigation on how melatonin interacts with ROR signaling and urge the melatonin research community for a paradigm shift in the direct interaction of melatonin toward RORs. The quest to identify nuclear receptors for melatonin in neuronal cells remains valid for the community to achieve.

**Supplementary Information:**

The online version contains supplementary material available at 10.1007/s12035-024-04395-y.

## Introduction

Physiological roles of melatonin have been associated with several cellular processes including circadian control, immunity, reproduction, oxidative defence mechanism, and aging [1–5]. Apart from its antioxidant and free radical scavenging properties, the effects of melatonin are mediated via signal transduction and its associated melatonin receptors including the membrane-bound melatonin receptors (MT1/2), which belong to the G protein-coupled receptor (GPCR) superfamily, the cytoplasmic melatonin receptor (MT3), and the nuclear ROR receptors. Recently, the structures of MT1 and MT2 have been elucidated by an X-ray free electron laser (XFEL) showing the interaction sites of melatonin agonists including ramelteon, agomelatine, and 2-phenylmelatonin [6, 7]. As of April 2024, nine entries of protein structures containing melatonin have been deposited in the Protein Data Bank (PDB) (Supplementary Table 1). Five of which are the cytoplasmic melatonin receptor (MT3) or known as the enzyme quinone reductase II (QR2) [8], emphasizing the antioxidant property of melatonin acting at least in part via the QR2. Additionally, melatonin has long been proposed to be a natural ligand of the nuclear retinoic acid receptor (RAR)-related orphan receptor β (RORβ) which is a member of the nuclear receptor (NR) superfamily [9–14]. RORs consist of three major isoforms in humans, including RORα, RORβ, and RORγ. In mammals, 17 variants of ROR proteins are encoded by *RORA*, *RORB*, and *RORC* genes with different alternative splicing sites. Among all ROR isoforms, RORβ is highly expressed and localized in the brain, especially in the suprachiasmatic nucleus, the pineal gland, and the retina, suggesting its role in regulating circadian rhythm [15].

No melatonin-bound RORβ structure has been reported to date, and only three rat RORβ structures have been deposited in the PDB (Supplementary Table 2). The reported RORβ isoforms were in complexes with stearic acids and all-trans-retinoic acids/synthetic retinoids in the LBD of RORβ. Retinoic acid (RA) was identified as the only natural ligand of RORβ, modulating the transcriptional activity of RORβ and RORγ but not RORα in a cell type-specific manner [16, 17]. The nuclear receptor co-activator proteins containing LXXLL motifs were found to be co-crystallized with RORs [16]. The binding of a ligand and co-activator protein is suggested to facilitate RORβ in adopting its active conformation and translocation to the nucleus for transcriptional activation of the target genes.

In the present study, we determined the potential binding site of melatonin on different isoforms of RORs. The homology model of human RORβ was generated by molecular modeling and employed as the receptor for molecular docking studies. We investigated the direct interaction of melatonin toward the LBD of RORβ using biophysical approaches. Finally, we investigated gene expressions of RORα, RORβ, and RORγ upon melatonin exposure using droplet digital polymerase chain reaction (ddPCR). Overall, this study aims to determine whether melatonin can directly bind to this nuclear receptor or whether it can impact the expression level of all ROR isoforms.

## Materials and Methods

### Sequence Analysis

Sequence alignment of RORα, RORβ, and RORγ was performed using Clustal OMEGA (RRID:SCR_001591). A structure-based sequence alignment was executed using PROfile Multiple Alignment with Local Structures and 3D constraints (PROMALS3D) (RRID:SCR_018161) analysis. PROMALS3D reveals the similarities and differences among the protein structures. Motifs discovery was conducted using motifFinder (https://www.genome.jp/tools/motif/). In addition to motifFinder, MEME Suite–motif-based sequence analysis tools (RRID:SCR_001783) were used to identify motifs. In both analyses, default parameters were used as per the developers’ recommendation.

### Molecular Modeling

The LBD of human RORβ was generated by homology modeling by SWISS-MODEL (RRID:SCR_018123) (https://swissmodel.expasy.org) using amino acid sequence (residues 210–450). Firstly, the best template was searched using BLAST (RRID:SCR_004870) (https://blast.ncbi.nlm.nih.gov/Blast.cgi) and HHBlits (RRID:SCR_010277) (https://toolkit.tuebingen.mpg.de/tools/hhblits) against the SWISS-MODEL template library. Overall, templates of 1991 structures were recruited, while the best homology match was selected based on its evolutionary related to the primary target sequence. The LBD of rat RORβ was used as a template structure which exhibits 98.02% sequence identity. The human RORβ models were generated based on the homology-conserved atomic coordinates by ProMod3, which enabled a re-modeling of insertions and missing sequences by its fragment library. The global and local model quality was assessed based on the highest global model quality estimate (GMQE) and the qualitative model energy analysis (QMEAN) scores.

### Molecular Docking and Molecular Dynamics Simulation

Crystal structures (PDB: 3KYT, 3B0W, and 1NQ7) were retrieved from the PDB database (RRID:SCR_012820), and all non-standard residues were removed. The protein structures were then optimized by adding H-atoms and charges. The missing atoms were fixed using Dock Prep [18]. The blind docking was performed using SwissDock [19, 20]. In Autodock Vina (RRID:SCR_011958), the local docking was performed in the defined area of the LBD of RORs. The grid center was set to 23.75 Å, 28.39 Å, and 10.18 Å for *x*-,*y*-, and *z*-axis. The grid size was adjusted to 22.00 Å in all three dimensions. The best binding mode of ligand–protein complexes was selected based on the lowest binding free energy (Gibbs free energy, ΔG_binding_) and FullFitness scores. The interacting residues in protein–ligand complexes were illustrated by a 2D plot using Discovery Studio Visualizer (BIOVIA, Dassault Systèmes).

For molecular dynamics (MD) simulation, the Desmond MD was utilized to further confirm the protein–ligand interactions. The simulation employed periodic boundary conditions, and protonation states at pH 7 were determined. One hundred fifty millimolar Na^+^ and Cl^−^ ions were introduced to establish equilibrium. The simulation employed the MTK barostat in an NPT ensemble for 1 µs at a constant temperature of 300 K and isotropic pressure of 1 bar. Explicit modeling of water molecules was conducted using TIP3P, with additional ions (Na^+^ or Cl^−^) included to neutralize protein charge. The stability of the complexes was assessed based on contact frequencies and root-mean-square deviation (RMSD). Trajectory analysis was performed using Schrodinger Maestro version 13.1.137 (Schrödinger, LLC).

### Protein Expression and Purification

The LBD of human RORβ containing amino acids 250–450 was expressed and produced in *E. coli* BL21 (DE3) using pET28a plasmid (GenScript). Cells were grown in LB medium at 37 °C until OD_600_ reached 0.6–0.8. Overexpression of the protein was induced at 16 °C using isopropyl β-D-1-thiogalactopyranoside (IPTG) at the final concentration of 1 mM. The purification protocol was performed following the previous study by Stehlin et al. [16]. Briefly, the pellets were lysed and sonicated using lysis buffer containing 20 mM Tris–HCl pH 8.6, 2 mM CHAPS, 100 mM NaCl, 2 mM β-mercaptoethanol (β-ME), and 20% glycerol. The supernatant was collected and loaded onto HisTrap HP column (GE Healthcare). The protein was eluted by a gradient of imidazole (0–1 M) and collected at 4 °C. The fraction containing protein was then loaded onto Superdex 75 10/300 column (GE Healthcare) and eluted using 20 mM Tris–HCl pH 8.6 containing 2 mM CHAPS, 100 mM NaCl, and 5 mM dithiothreitol (DTT). The freshly purified protein was collected for protein–ligand interaction experiments. The SRC-1 peptide used for biophysical interaction studies was synthesized by GenScript. The sequence of SRC-1 peptide is RHKILHRLLQEGSPS and freshly prepared into solution before performing assays.

### Differential Scanning Fluorometry

Differential scanning fluorometry (DSF) was performed using freshly purified RORβ in gel filtration elution buffer. Each 25-µL assay contained 50 µM RORβ, 10 × SYPRO Orange (Sigma-Aldrich), and various concentrations of melatonin (0, 100, 200, 500, 1000, and 2000 µM). DMSO at 0.4% was used as a control for the assay. DSF of the LBD of RORβ with SRC-1 peptide was performed at concentrations of 50, 100, 150, 200, 250, and 500 µM in the presence or absence of 1 mM melatonin. DSF assay was performed with 3 biological replicates using 3 batches of freshly prepared RORβ each in quadruplicate. The assay was prepared in a 96-well PCR plate (Bio-Rad #MLL9601) and sealed with adhesive film (Bio-Rad #MSB1001). The fluorescent signal was recorded using Bio-Rad CFX96 real-time PCR instrument using the following settings: melting curve analysis, FRET channel, 25–95 °C with 0.5 °C incremental step, and 30 s incubation for each step before signal capture. Protein was allowed to incubate within the complete reaction setting for 30 min before the DSF analysis. The apparent melting temperature (Tma) was calculated using DSFworld web application (https://gestwickilab.shinyapps.io/dsfworld/).

### Isothermal Titration Calorimetry

Isothermal titration calorimetry (ITC) experiments were carried out in a buffer of 20 mM Tris–HCl pH 8.6 containing 2 mM CHAPS, 100 mM NaCl, and 5 mM dithiothreitol (DTT). Protein was dialyzed against this buffer overnight before the experiment. Subsequent dilutions of protein (the LBD of RORβ) and ligands (SRC-1 peptide or melatonin) used in the ITC assay were performed using the remaining dialysis buffer. Generally, ligands at a concentration of around 1–5 mM were titrated against 50–100 µM RORβ in the Malvern MicroCal PEAQ-ITC instrument. When ligands were dissolved in DMSO, an appropriate concentration of DMSO (0.5–1% v/v) was added to the titrate solutions. Data were analyzed using MicroCal PEAQ-ITC analysis software. The titration of an initial injection (0.2 µL) was discarded during data processing.

### Gene Expression Analysis

SH-SY5Y cells (RRID:CVCL_0019) were treated with various melatonin concentrations for 24 h at the final concentration of 0.01, 0.1, 1, and 10 µM. Total RNA was extracted from SH-SY5Y cells using TRIzol reagent (Invitrogen) according to the manufacturer’s instructions. Subsequent cDNA was synthesized using the Reverse Transcription Kit (Applied Biosystems). Droplet digital polymerase chain reaction (ddPCR) was performed on the Bio-Rad QX200 ddPCR system (Bio-Rad) following the manufacturer’s instruction. Droplets were generated from reactions containing 50 ng of cDNA using ddPCR EvaGreen Supermix (Bio-Rad). The primers used in this study are shown in Table 1.

**Table 1:** Primer sequences used in the study

Gene	Forward primer	Reverse primer
*hRORA(alpha)*	CTTCTTTCCCTACTGTTCGTTC	GCTCTTCTCTCAAGTATTGGC
*hRORB(beta)*	CATGCAAAATTTGTGGCGATAA	CCCTTGCAGCCTTCACATGT
*hRORC(gamma)*	GCAAAGAAGACCCACACCTC	CACCCCTCACAGGTGATAA
*hGAPDH*	GGCCTCCAAGGAGTAAGACC	AGGGGAGATTCAGTGTGGTG

Quantitative PCR (qPCR) was also carried out to assess the effect of 1 µM luzindole, a melatonin receptor MT1/MT2 antagonist, on melatonin-induced RORβ expression in SH-SY5Y cells. In this experiment, 1 µM luzindole was incubated for 30 min prior to 10 µM melatonin treatments for 24 h [21]. RNA was extracted by TRIzol reagent (Invitrogen) according to the manufacturer’s instructions. Then, 100 ng of cDNA was used to prepared PCR reactions using Luna® Universal qPCR Master Mix (New England Biolabs). The reaction protocol included an initial denaturation step at 95 °C for 2 min, followed by 40 cycles consisting of 95 °C for 15 s, 60 °C for 15 s, and 68 °C for 20 s. Glyceraldehyde-3-phosphate dehydrogenase (GAPDH) was employed as the internal control for normalization, and relative mRNA expression levels were analyzed using the 2^−ΔΔCt^ method [22].

The data were presented as mean ± SEM (standard error of the mean). Statistical significance was determined using analysis of variance (ANOVA) followed by Tukey’s post hoc test, with significance set at *P* < 0.05.

## Results

### Human RORs Sequence Analysis Revealed DNA Binding Motifs and Ligand Binding Domains

**Fig. 1:**
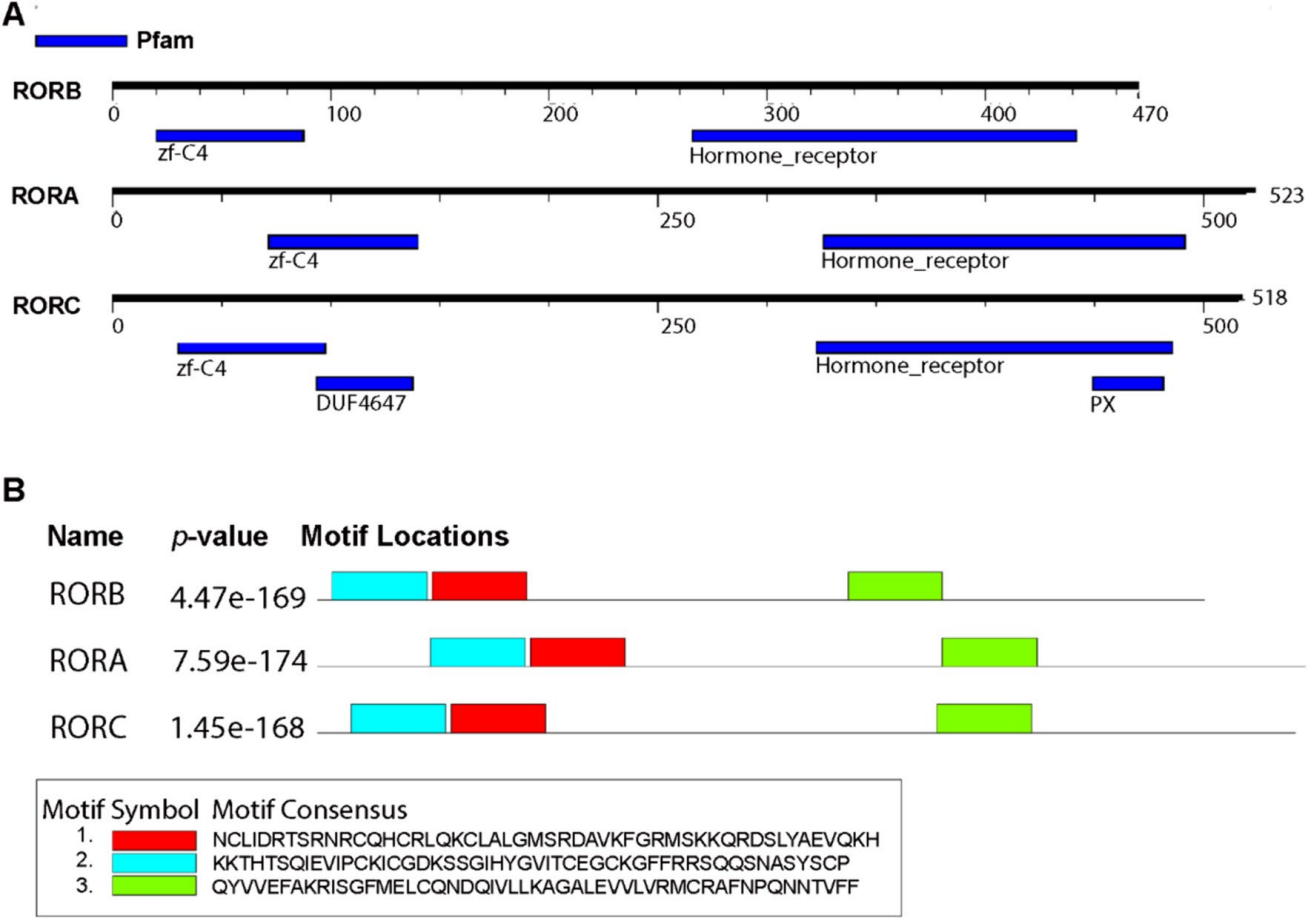
Schematic diagrams of motifs identified along RORA(α), RORB(β), and RORC(γ). **A** Motifs discovered using motifFinder (https://www.genome.jp/tools/motif/). The motifFinder detected four motifs: zinc finger C4 type, a ligand-binding domain of nuclear hormone receptor, the domain of unknown function, and the PX domain. The motifs are represented by blue rectangular boxes. Black solid line represents corresponding ROR proteins, and its length denotes by a residue scale. **B** The MEME suite identified three common motifs. Each motif is color coded; accordingly, motif 1: red (zinc finger, C4 type); motif 2: blue (C1 domain); motif 3: green (the LBD of nuclear hormone receptor). The *p*-value indicates the likelihood of randomly observing the identified motif. A lower *p*-value indicates that the observed motif is less likely to occur by chance

The *Homo sapiens* protein sequences of RORα, RORβ, and RORγ were retrieved from the UniProt database. RORα, RORβ, and RORγ contain 523, 470, and 518 amino acids, respectively. BLAST information derived from the Swiss-Prot confirmed these sequences belong to the retinoic acid receptor family. Subsequently, these three protein sequences were subjected to PROfile Multiple Alignment with Local Structures and 3D constraints (PROMALS3D) analysis. PROMALS3D analysis identified 208 conserved amino acids (Supplementary Fig. 1). A closer look at the conserved regions revealed these invariant amino acids spanning along the alpha–beta secondary structures. Motifs discovery was conducted using motifFinder (https://www.genome.jp/tools/motif/) and Multiple Em for Motif Elicitation (MEME) suite. The motifFinder detected four motifs corresponding to zinc finger C4 type, ligand-binding domain, the domain of an unknown function (DUF4647), and the Phox homology (PX) domains (Fig. 1A). Interestingly, the domains of unknown function and the PX domain were unique to the RORγ. Additionally, MEME revealed three common motifs across RORα, RORβ, and RORγ (Fig. 1B). Motif 1 represents a C4-type zinc finger motif which is well-characterized in the nuclear hormone receptor family employing four cysteines to coordinate zinc ions with. This motif is thus responsible for binding to target genes. Motif 2 also contains zinc finger motifs comprising a C1 domain capable of binding to two atoms of zinc ions. It features a short cysteine-rich domain. Lastly, motif 3 is a ligand-binding domain typically found among nuclear hormone receptors.

### Molecular Docking Revealed Potential Binding of Melatonin on Rat RORβ

**Fig. 2:**
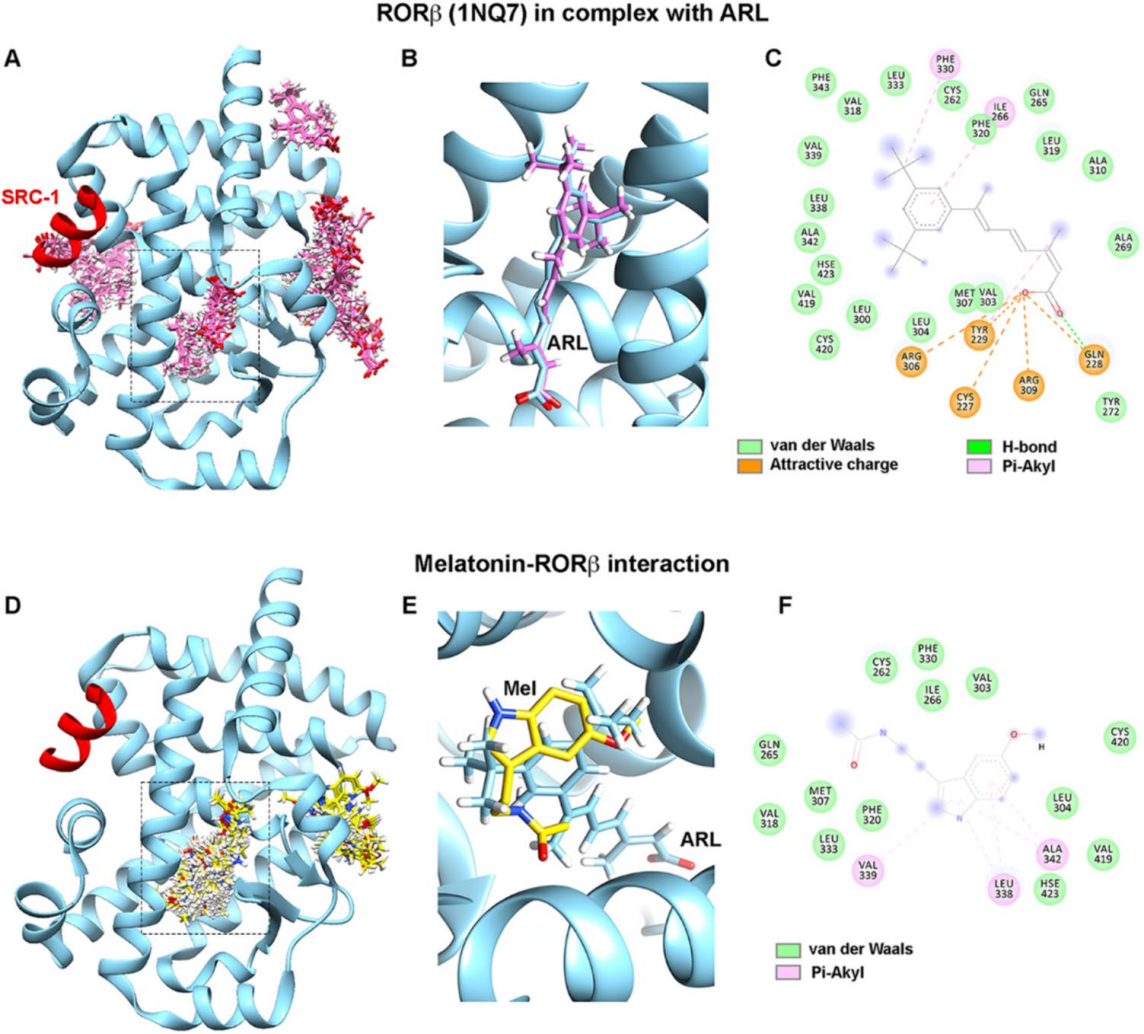
Prediction of melatonin binding sites and intermolecular forces on rat RORβ. **A** Molecular docking by SwissDock was validated by re-docking the ligand, ARL (pink sticks), to the receptor molecule (PDB: 1NQ7). Four target cavities consisting of 34 binding clusters were predicted. **B** The most favorable binding pose (pink sticks) of the ARL-RORβ complex is shown superimposed with the ARL position from the crystal structure (blue stick). **C** The protein–ligand interactions are illustrated by the 2D plot. **D** The predicted melatonin binding sites (yellow sticks) on RORβ consisted of two binding cavities of 42 clusters. **E** The best pose of melatonin (yellow sticks) is shown superimposed with the ARL position from the crystal structure (blue stick). **F** Melatonin interacting residues are demonstrated in the 2D plot. The ligand binding pocket derived from the crystal structure is marked by dashed rectangles. Ligands are depicted as sticks with oxygen, hydrogen, and nitrogen atoms colored in red, white, and blue, respectively

Due to its high resolution (1.5 Å) compared to other reported structures, the crystal structure of rat RORβ in complex with 7-(3,5-ditert-butylphenyl)-3-methylocta-2,4,6-trienoic acid (ARL) was chosen to perform molecular docking (Supplementary Table 2). The docking protocol was initially verified by re-docking the receptor protein with ARL, the only known ligand for RORβ (PDB: 1NQ7). Using SwissDock, 34 possible binding clusters containing 257 poses were predicted to be potential ARL interaction sites, which consisted of four target cavities (Fig. 2A). The best ARL-RORβ interaction site was precisely predicted at the similar position compared to the ligand-binding site validated by X-ray crystallography (Fig. 2B). The lowest binding free energy (Gibbs free energy, ΔG_binding_) and FullFitness scores of − 11.78 and − 1350.85 kcal/mol were calculated. The residues involving in protein–ligand interactions showed that the ARL binding position was substantially preserved through van der Waals forces and some electrostatic interactions, including charge-charge attraction, Pi(π)-alkyl, and H-bonding (Fig. 2C). Using rat RORβ as the protein receptor, the predicted melatonin-RORβ binding sites consisted of 42 clusters with 252 ligand binding poses containing within two possible binding pockets (Fig. 2D). The best pose of melatonin binding sites was selected based on the most favorable binding energy (ΔG_binding_, − 7.22 kcal/mol) and FullFitness scores (− 1325.53 kcal/mol) (Fig. 2E, F). Melatonin primarily maintained in the binding pocket by van der Waals forces and three Pi(π)-alkyl anchoring the indole ring of melatonin with Leu338, Val339, and Ala342 (Fig. 2F). The best melatonin-RORβ binding site was predicted at the same binding site of ARL, albeit melatonin occupied a lesser surface area of 408 Å^2^ compared to that of ARL (632 Å^2^) due to the smaller size of the molecule. (Fig. 2E). In addition, the binding energy of melatonin to rat RORβ was confirmed using AutoDock Vina to perform local docking. In AutoDock Vina, melatonin was calculated to bind with RORβ with a ΔG_binding_ of − 7.40 kcal/mol, and the binding position was in the similar binding pocket as predicted by the blind docking using SwissDock. We also docked melatonin to the well-characterized MT1 (PDB: 6ME5) and MT3 receptor (PDB: 2QWX) as a comparator. The results showed that the estimated binding energies of melatonin on MT1 and MT3 were − 7.52 kcal/mol and − 7.36 kcal/mol, respectively, which are comparable to the binding energy with RORβ. The binding site of melatonin from docking prediction and the agomelatine-MT1 interaction site solved by XFEL are illustrated in Supplementary Fig. 2A, B.

### Molecular Docking Revealed Potential Melatonin Binding to All Human RORs

**Fig. 3:**
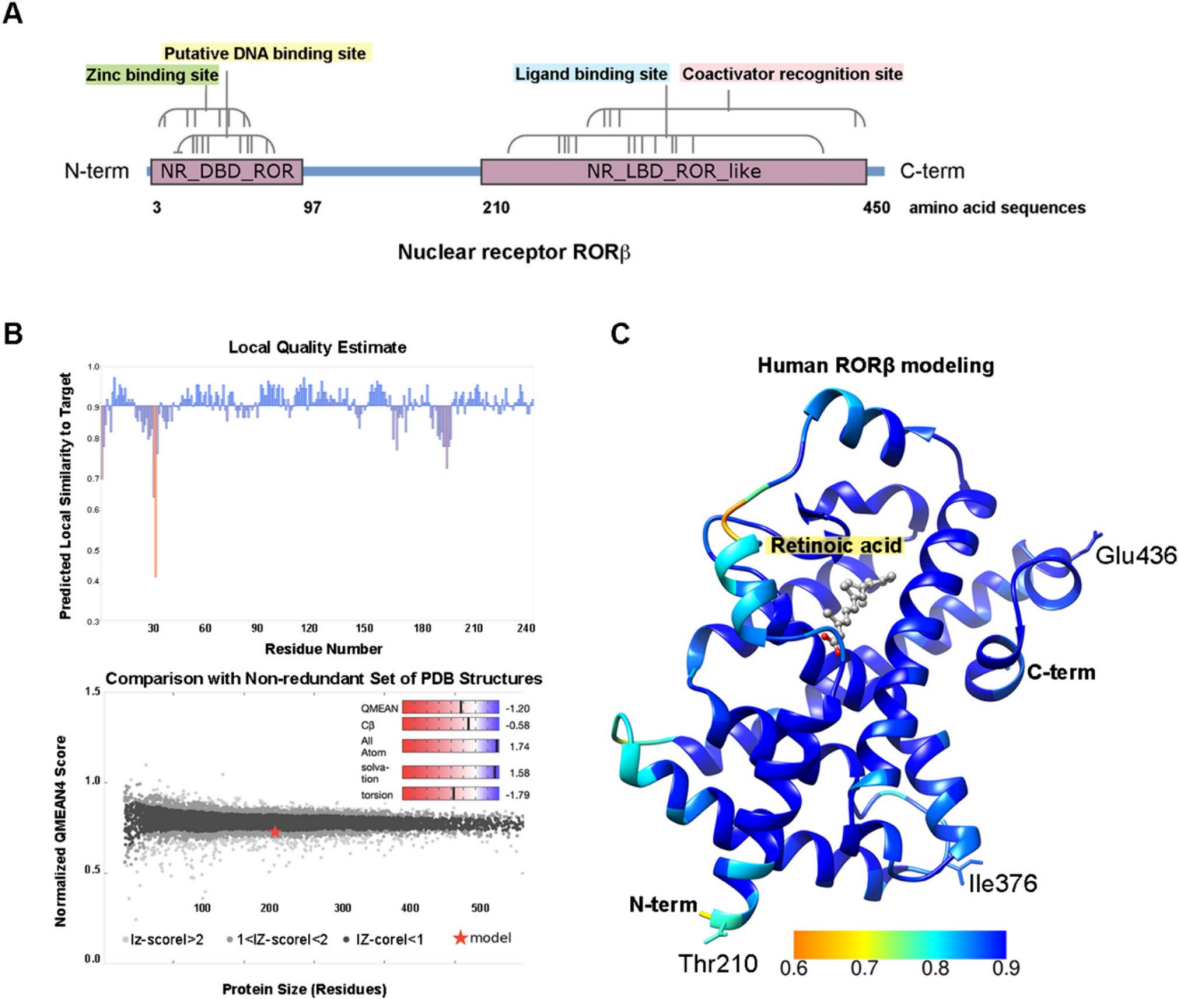
Molecular modeling of the human RORβ ligand binding domain using homology modeling. **A** The full length of human RORβ with motifs and domain. The ligand binding domain contains residues 210–450. **B** Local quality estimate and normalized QMEAN score were estimated per residue. **C** The LBD of the human RORβ structure was built by homology modeling, using the rat RORβ as a template (PDB: 1N4H). The rat RORβ comprises sequence identity of 98.02% to the human RORβ, while the residues that are not conserved are shown including Thr210, Ile376, and Glu436. Ribbons colored in blue represent high degree of confidence (about 90%). The position of the LBD pocket is occupied by a ligand, retinoic acid, derived from the crystal structure of rat RORβ co-crystalized with retinoic acid. Ligands are depicted as sticks with oxygen and carbon atoms colored in red and grey, respectively

To compare a potential melatonin binding site on different isoforms of human RORs, firstly, the structure of human RORβ was generated using homology modeling, as currently there is no crystal structure of human RORβ available. Human RORβ contains 459 amino acids, while the LBD is at residues 210–450 (Fig. 3A). The human LBD amino acid sequence was searched in SWISS-MODEL template library using BLAST and HHBlits. Overall, templates of 1991 structures were found. The best match was selected based on its evolutionarily related to the primary target sequence. The template (PDB: 1N4H) containing a sequence identity of 98.02% belongs to the rat RORβ. The model was built using target-template alignment by ProMod3. The model quality was assessed based on the highest GMQE and QMEAN scores of 0.93 and − 1.20, respectively, indicating that the model structure was of good quality compared to the experimental structure of similar size (Fig. 3B, C). Three amino acids of the human RORβ ligand-binding domain differ from the rat RORβ structure. However, these differences did not affect the receptor secondary structure (Fig. 3C).

**Fig. 4:**
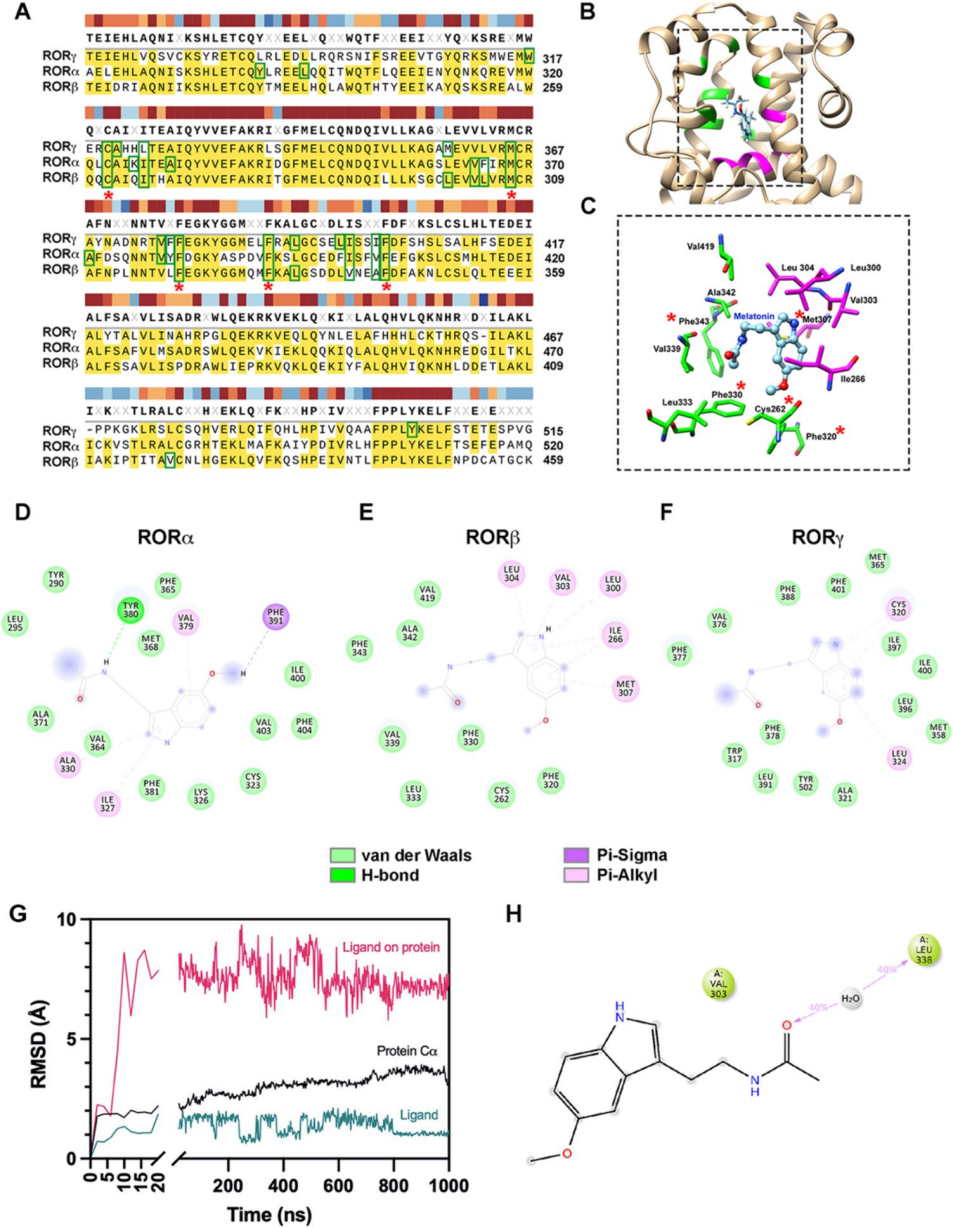
Prediction of melatonin binding sites and intermolecular forces on human RORs. **A** Sequence alignments of the LBD of human RORα/β/γ highlight amino acid conservation among receptor isoforms. The residues involved in melatonin interaction are in green boxes and marked with asterisks toward their conservation among three isoforms. **B**, **C** The homology model of the human RORβ LBD was carried out with molecular docking to predict melatonin binding sites. The LBD binding pocket is shown in rectangle. Melatonin is depicted as sticks with oxygen, carbon, and nitrogen shown in red, cyan, and blue, respectively. The residues colored in green interact with the ligand by van der Waals attractive forces, while the residues colored in pink form Pi(π)-Alkyl interaction to the ligand. **D**, **E**, **F** The best melatonin-ROR interaction sites of human RORα/β/γ and their intermolecular forces are depicted by the 2D plots. ΔG_binding_ =  − 7.25, − 7.15, and − 7.14 kcal/mol and FullFitness scores =  − 1387.10, − 1151.36, and − 1411.89 kcal/mol correspond to RORα, RORβ, and RORγ, respectively. **G** The root-mean-square deviation (RMSD) was used to calculate the average change in displacement of a particular set of atoms for each frame relative to a reference frame. **H** The 2D plot illustrates the interaction between RORβ LBD and melatonin obtained from a 1-µs molecular dynamics simulation. The diagram shows specific ligand atom interactions with protein residues of the chosen trajectory from 0.00 through 1000 ns which interacted for more than 30.0% of the total simulation time

To predict melatonin binding sites, the homology model of human RORβ and the crystal structures of RORα/γ were used to perform molecular docking. The best melatonin-ROR interaction sites of human RORα/β/γ were selected based on the lowest protein–ligand binding energy. The residues predicted to involve with melatonin binding sites are shown in Fig. 4A. The LBD of human RORα shares 60.40% and 52.42% amino acid sequence identity to human RORβ and RORγ, respectively, while RORβ and RORγ share only 49.19% sequence identity. Nearly most predicted interacting residues are conserved in all RORs, except Ala342 in RORβ that is substituted by Val in RORα and Ile in RORγ (Fig. 4A). Five residues predicted to interact with melatonin in all isoforms are shown to be fully conserved among RORα/β/γ isoforms, including Cys262, Met307, Phe320, Phe330, and Phe343, suggesting that melatonin could likely be a ligand for all RORs (Fig. 4A–C). Comparable ΔG_binding_ of − 7.25, − 7.15, and − 7.14 kcal/mol were calculated for RORα, RORβ, and RORγ, respectively. The predicted intermolecular forces contributing to ROR-melatonin interaction form the hydrophobic pocket and allow melatonin to sit in the LBD primarily arising solely from van de Waals attractive forces. The protein–ligand interactions of RORα/β/γ and melatonin are illustrated by 2D plots (Fig. 4D–F).

However, the molecular dynamics (MD) simulation of the melatonin-RORβ complex revealed only weak interactions between melatonin and the LBD of RORβ. Throughout the 1 µs MD simulation, melatonin exhibited unstable binding with the LBD, primarily interacting directly with Val303 and indirectly with Leu338 (Fig. 4G, H). Notably, melatonin did not show any interactions with the two conserved residues, Arg306 and Arg309, which are crucial for ligand binding in the ROR ligand-binding pocket [17]. Therefore, further biophysical assays are required to draw a clear conclusion regarding the involvement of RORβ as a nuclear receptor for melatonin.

### Biophysical Assays Could Not Confirm Binding Interaction Between the LBD of RORβ and Melatonin

**Fig. 5:**
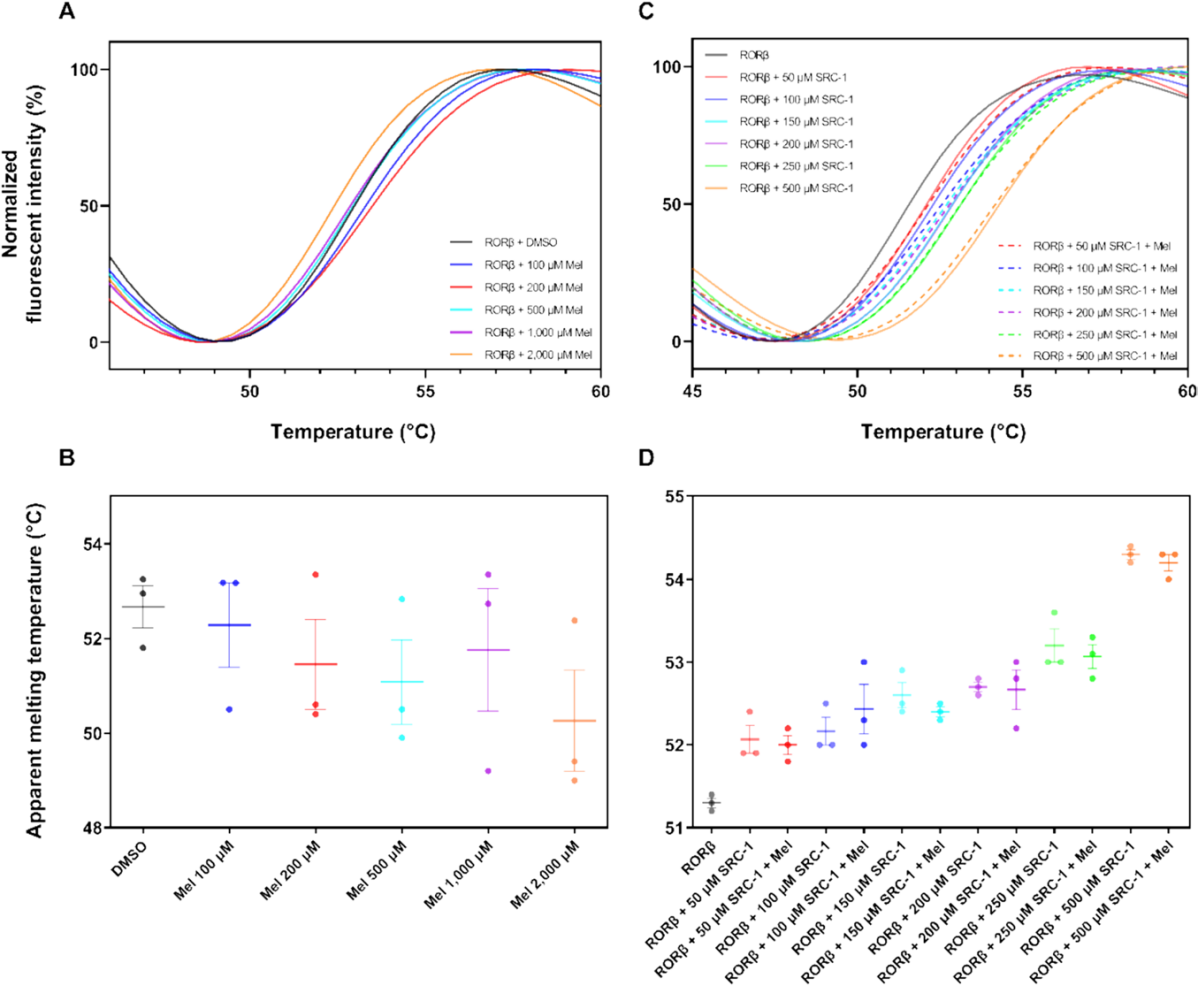
Differential scanning fluorometry results. **A** Normalized fluorescent intensities were plotted against temperature for the LBD of RORβ treated with 100, 200, 500, 1000, and 2000 µM melatonin. **B** Mean of apparent melting temperature (Tma) of the LBD of RORβ treated with 100, 200, 500, 1000, and 2000 µM melatonin. **C** Normalized fluorescent values were plotted against temperature for the LBD of RORβ treated with 50, 100, 150, 200, 250, and 500 µM SRC-1 peptides, in the presence or absence of 1 µM melatonin. **D** Mean of apparent melting temperature (Tma) of the LBD of RORβ treated with 50, 100, 150, 200, 250, and 500 µM SRC-1 peptide, in the presence or absence of 1 µM melatonin

**Fig. 6:**
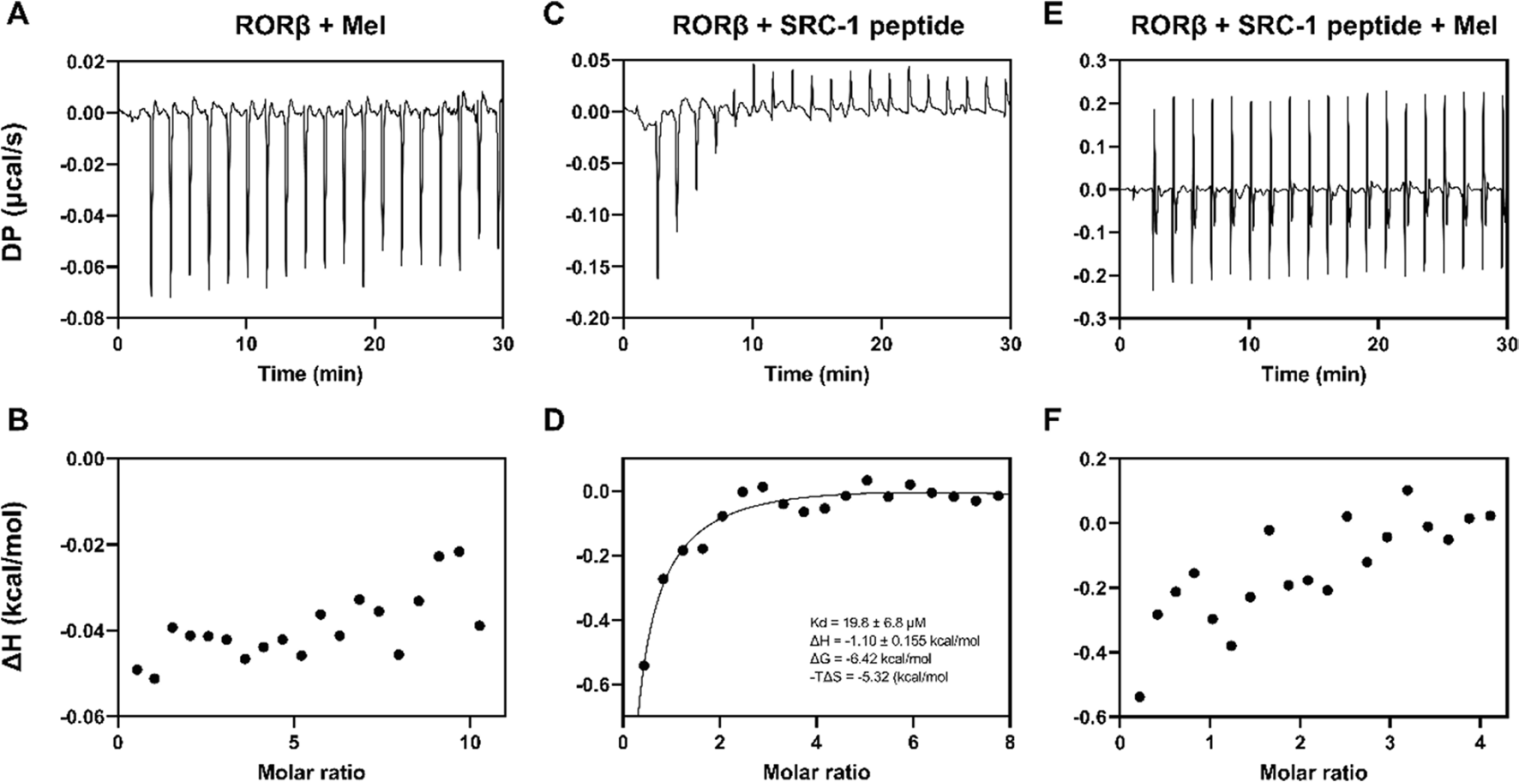
Isothermal calorimetry (ITC) results. **A**, **B** The LBD of RORβ was titrated with 5 mM melatonin. **C**, **D** The LBD of RORβ was titrated with 2 mM SRC-1 peptide. **E**, **F** The LBD of RORβ was pre-incubated with 500 µM of SRC-1 peptide and titrated with 1 mM melatonin. Top panel shows raw data of heat change over time. Bottom panel shows enthalpy change per mole of ligand against ligand:protein molar ratio. Measurement from the first injection was excluded from all analyses

We further investigated the direct interaction of the neuron-specific RORβ and melatonin in solution using biophysical tools. Differential scanning fluorometry (DSF) assay was applied to initially explore the potential interaction between the LBD of RORβ and melatonin. In our assay, melatonin did not shift the apparent melting temperature (Tma) of the LBD of RORβ significantly (Fig. 5B and D). The LBD of RORβ treated with 0.4% DMSO has a Tma of 52.67 ± 0.44 °C, whereas the LBD of RORβ treated with melatonin, at all concentration tested, has a Tma in the range of 50.28 ± 1.06–52.28 ± 0.89 °C. As expected, SRC-1 peptide increased Tma of the LBD of RORβ in dose-dependent fashion from 51.30 ± 0.05 to 54.30 ± 0.05 °C at 50 to 500 µM SRC-1 (Fig. 5D). Although, in the presence of SRC-1 peptide, melatonin could not alter the Tma of the LBD of RORβ (Fig. 5D). As the shift in the Tma was subtle (< 5 SD of the control Tma) in all melatonin treated conditions, we could not draw any clear conclusion from the experiment. Therefore, the more sensitive and detailed experimentation by isothermal titration calorimetry (ITC) was performed to experimentally elucidate the interaction between the two. ITC result could confirm the interaction between SRC-1 peptide and the LBD of RORβ (Fig. 6C, D) with a K_D_ of 19.8 µM, and the reaction was exothermic (ΔH =  − 1.1 kcal/mol). The binding was thermodynamically favorable with a ΔG value of − 6.42 kcal/mol. However, ITC confirmed no interaction between the LBD of RORβ and melatonin in all of our settings, with or without SRC-1 peptide (Fig. 6A, B, E, and F).

### Melatonin Substantially Altered ROR Gene Expressions

**Fig. 7:**
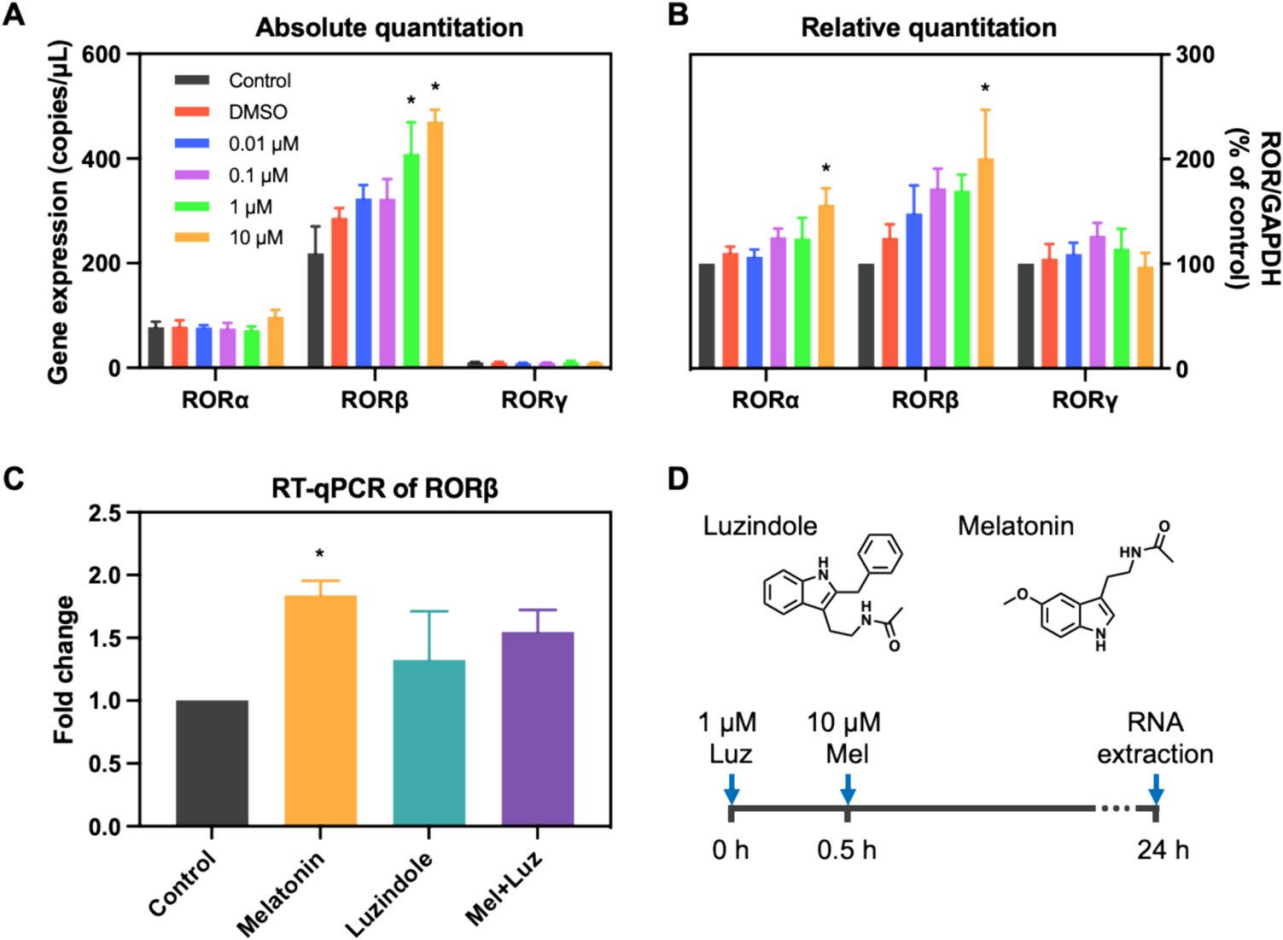
The effect of melatonin on RORα, RORβ, and RORγ mRNA expression levels. Cells were incubated with various concentrations of melatonin for 24 h at the final concentration of 0.01, 0.1, 1, and 10 µM. ddPCR was used to determine the absolute expression levels of RORα/β**/**γ gene expressions (**A**) and calculated for relative expression (**B**). RT-qPCR of melatonin treatment in the presence of MT1/MT2 antagonist luzindole (**C**) as depicted in **D**. The copies of gene and data were represented as the means ± SEM. Experiments were performed with at least triplicates. Asterisk (*) denotes *p* < 0.05 compared to the control

We finally investigated whether melatonin influenced RORs gene expression levels using ddPCR in human neuroblastoma SH-SY5Y cell lines. In human, there are three primary ROR isoforms, including RORα, RORβ, and RORγ. RORβ is abundantly expressed in the brain, particularly in the suprachiasmatic nucleus, the pineal gland, and the retina, suggesting a function in the control of circadian rhythms. To investigate the effect of melatonin on ROR expression, SH-SY5Y cells were treated with various concentrations of melatonin at the final concentration of 0.01, 0.1, 1, and 10 µM. Our results showed that RORβ is highly expressed in the SH-SY5Y neuroblastoma cell line, compared to RORα and RORγ isoforms (Fig. 7). As expected, the expression of RORγ was very limited in SH-SY5Y cells (Fig. 7). Administration of melatonin at 10 µM significantly increased the mRNA expression levels of RORα and RORβ (*p* < 0.05). However, this effect of melatonin was not observed in RORγ isoform. Thus, our results suggest that melatonin may, to some extent, affect the brain-enriched ROR isoforms indirectly by upregulating the mRNA expression levels. We further investigated whether the effect of melatonin on the neuron-specific RORβ is mediated via the membrane receptors, MT1 and MT2, using the melatonin receptor antagonist luzindole. One µM luzindole was incubated for 30 min prior to melatonin treatment at 10 µM for 24 h. RT-qPCR showed that elevated RORβ substantially persisted in the luzindole pre-treatment. This result suggests that the upregulation of RORβ is unlikely modulated via MT1 and/or MT2 signal transductions.

## Discussion

Physiological roles of RORs involve several cellular processes including the regulation of development, circadian rhythm, metabolic pathway, and immunity [23]. The connection of melatonin and ROR physiological effects involve circadian gene regulation. Melatonin and RORs are considered the circadian oscillators regulating clock and clock-controlled gene transcription [24]. RORs bind to the ROR response elements (ROREs) on target genes [25] and activate the transcription of several circadian genes including *Bmal1*, *NPAS2*, and *REV-ERBα* [26–28]. The study in non-neuronal HeLa cells found that RORα and RORγ upregulate the transcription of the circadian transcription factors, *Bmal1*, up to 16 and fivefold, respectively [29]. The functional dysregulation of RORs has been reported in many diseases including multiple sclerosis, autoimmune diseases, metabolic diseases, cancer, and asthma [30].

The hypothesis of melatonin nuclear receptor was proposed since melatonin has been found to localize in the nucleus [31, 32]. Afterward, several studies demonstrated that RORs, a member of the nuclear receptor superfamily, were the nuclear receptor for melatonin. Recently, some emerging data opposing this idea has been proposed suggesting that melatonin may not a ligand for the putative nuclear RORs (for a review, see ref. [33]). In 1994, melatonin was firstly reported to bind directly with RORβ and stimulate its transcriptional activity [9]. However, in 1997, the authors requested the publisher for additions and corrections of the original paper that they could not reproduce the experimental part concerning the direct interaction of melatonin and RORβ [34]. Later, few studies reported that melatonin failed to activate RORβ transcriptional activity in Neuro2A cells and could not inhibit RORα/γ transcriptional activity in Chinese hamster ovary cells overexpressing with RORα/γ vectors [35, 36]. In addition, Slominski et al. (2016) reported that melatonin is not a natural ligand of RORα due to its lower affinity, respective to cholesterol and other fatty acid-derived compounds [36, 37].

On the contrary, several studies claiming that melatonin acts via its nuclear receptors have been proposed up until now. Multiple lines of evidence have shown that all isoforms of RORs are the mediators conveying melatonin effects, as proven by several models of studies ranging from RORα/β/γ-knock out models, specific inhibitors, or siRNA blockage [13, 14]. Melatonin has been suggested to inhibit hepatic stellate cell (HSC) activation through RORα, as melatonin membrane receptors (MT1/2) are not expressed in this cell line [12]. Furthermore, the RORα antagonist (SR1001) could also block the effect of melatonin on suppressing HSC activation [12]. The study carried out in human Jurkat T-cells showed co-localization of melatonin with RORα in the nucleus, emphasizing its role as a ligand for RORs [11]. Another study proposed that melatonin treatment enabled the restoration of RORα protein content in the nucleus and enhanced Sirt1 mRNA expression, resulting in an inhibition of inflammatory responses in septic mice [10]. Additionally, these effects of melatonin could not convey in the septic RORα functional knockout mice [10]. Furthermore, functional disruption of RORβ resulted in an alteration of animal behavior related to circadian rhythm dysregulation and the degeneration of the retina in postnatal development [38]. The expression of RORβ was tightly regulated by the biological clock showing its highest expression at night in the pineal gland and the retina, major RORβ expressing areas [15]. Moreover, the study using radioactive labeling melatonin showed that the binding of melatonin to its membrane and nuclear receptors in the thymus and spleen could be blocked by their specific inhibitors [39]. The discordance in the findings regarding the nuclear effects of melatonin led to a broad confusion among the scientists in the field of melatonin research. Nonetheless, solid evidence of direct interaction between RORs and melatonin has not yet been established. We, therefore, investigated whether melatonin can directly bind with the LBD of RORs, in particular RORβ, by exploiting computational and biophysical approaches.

In this study, we initially employed protein sequence analysis, molecular modeling, and molecular docking to study the potential binding property of melatonin on human RORs. The sequence analyses showed that the LBD of human RORα/β/γ isoforms shared 50–60% sequence identity, despite that the secondary structures of these isoforms are conserved (Supplementary Fig. 1). Further analysis showed that all human RORs contained an LBD and DNA binding motifs. Interestingly, the domains of unknown function and the PX domain were uniquely identified in the RORγ (Fig. 1A). Physiological roles of the PX domain involve membrane phosphoinositide binding anchoring the protein toward organelle membrane lipids [40], suggesting that RORγ may possess other unknown/diverse functions in cellular processes. Other motifs found in all RORs were C1- and C4-type zinc finger domains and hormonal ligand-binding domains (Fig. 1A, B). These motifs and domains suggested that RORs are likely nuclear receptors of some types which are capable of regulating gene expression. These findings are in accordance with previous reports on RORs as transcription regulators [25–28]. We further analyzed the possible binding sites for melatonin on the RORβ using rat and human RORs as the model. The docking results suggested that melatonin was potentially able to bind the LBD of all RORs with moderate predicted ΔG_binding_ in all ROR isoforms (− 7.14 to − 7.25 kcal/mol). Furthermore, all isoforms of these nuclear receptors shared a potential binding site and conserved residues for predicted melatonin interaction (Fig. 4A–C). However, the intermolecular forces of melatonin and RORs largely arose from van de Waals attractive forces, and no polar interaction was observed. Based on ΔG_binding_ and FullFitness scores, ARL showed more favorable binding to the LBD of RORβ. However, the favorable scores of Fitness function (FullFitness) concerning ligand binding modes (i.e., position, orientation, and conformation) of these two compounds were comparable. Considering the structure of ARL (C_23_H_32_O_2_, 57 atoms), it contains a larger molecular size compared to that of melatonin (C_13_H_16_N_2_O_2_, 33 atoms). Typically, a bigger molecule occupies more space and has more atomic elements to interact with the protein receptors by creating van der Waals attractive forces. To elucidate this precaution, we compared the estimated binding energy of melatonin-RORβ to those predicted using MT1 (PDB: 6ME5) and MT3 receptor (PDB: 2QWX) as the known target references. The predicted binding energies were commensurate with that of melatonin with its well-characterized MT1 and MT3 at − 7.52 and − 7.36 kcal/mol, respectively. Taken together, these docking experiments using rat RORβ and human RORs suggested that melatonin was potentially able to bind RORβ. We therefore opted to study the direct interaction between the LBD of RORβ and melatonin experimentally using biophysical approaches, DSF and ITC.

DSF is a medium-throughput biophysical assay based on the accessibility of the hydrophobic dye SYPRO Orange to the protein. The assay will provide insight into the thermal stability of the protein as reflected by the apparent melting temperature, Tma. A protein with high thermal stability will have high Tma. The thermal stability of a protein is affected by many factors, one of which is the ligand binding [41]. DSF analysis of the LBD of RORβ with various concentrations of melatonin showed no significant Tma shift from the control, although a slight decrease in Tma was observed (Fig. 5A, B). We were aware that the LBD of RORβ might not be in a preferred conformation as there is evidence showing that the LBD of rat RORβ adopted its active conformation upon binding with co-activator peptide SRC-1 [16]. Therefore, we further analyzed the Tma of the LBD of RORβ in the presence of various concentrations of SRC-1 co-activator peptide. As expected, SRC-1 peptide could stabilize the LBD of RORβ as a significant shift in Tma was observed (Fig. 5C, D). However, melatonin did not alter the Tma of the LBD of RORβ, albeit in the presence of SRC-1 at all concentrations (Fig. 5C, D). As the DSF result did not support the computational finding, we sought for another biophysical assay that is more robust for interaction elucidation, isothermal titration calorimetry (ITC), as it is possible that true binding may not affect thermal stability of the protein, especially for the low to moderate affinity binders [42]. ITC results agreed with the finding from DSF assay. No interaction could be detected between the LBD of RORβ and melatonin with or without SRC-1 peptide (Fig. 6A, B, E, and F), while a moderate interaction with a K_D_ of 19.8 µM could be calculated for the LBD of RORβ and SRC-1 co-activator peptide, and the binding is thermodynamically favorable (Fig. 6C, D). Our data showed that the human RORβ isoform could not directly bind with melatonin as confirmed by both biophysical techniques even in the presence of co-activator peptide SRC-1.

Overall, we found that molecular docking, although useful for predicting ligand binding modes and affinities computationally, may not always align with experimental data, particularly due to its lack of the physiological environment. Additionally, DSF assesses changes in protein stability upon ligand binding rather than direct binding affinity. In contrast, ITC directly measures heat changes upon ligand binding, providing accurate thermodynamic parameters including K_D_ values in the nanomolar to micromolar range [43]. Therefore, we employed a complementary approach using these techniques to investigate the direct molecular interaction of RORβ and melatonin, acknowledging the unique characteristics and limitations of each method. However, we could not entirely rule out the possibility that RORs and melatonin are somehow related as many reports have shown the association between the two. We thus investigated the effect of melatonin on RORs gene expression in SH-SY5Y neuroblastoma cell line. Our RT-ddPCR gene expression analysis showed an increased expression of neuron-enriched RORs, RORα, and RORβ, upon melatonin treatment of various concentrations for 24 h, while very stably low RORγ transcript was detected at all concentrations tested. Although the increasing trend was observed, only treatments with higher concentrations (1 and 10 µM) of melatonin significantly affected the level of RORα and RORβ (Fig. 7). Furthermore, the upregulation of RORβ mRNA levels was not completely abolished by luzindole pre-treatment. It is commonly accepted that melatonin binds and activates membrane receptors MT1 and MT2 at picomolar to nanomolar concentrations, thereby modulating downstream signaling pathways [44, 45]. At micromolar to millimolar range, melatonin intrinsically exhibits potent antioxidant and free radical scavenging properties [46, 47]. Therefore, there are several reasons to believe that the elevated expression level of RORβ is not wholly through the MT1 and/or MT2 receptors and cascading signal transduction. Firstly, mRNA level of RORβ should be affected by melatonin at nanomolar concentration. Secondly, luzindole pre-treatment should restore RORβ expression levels to the baseline. These findings suggest that the relationship and interplay between melatonin, RORs, and other target genes are much complicated than previously anticipated.

**Fig. 8:**
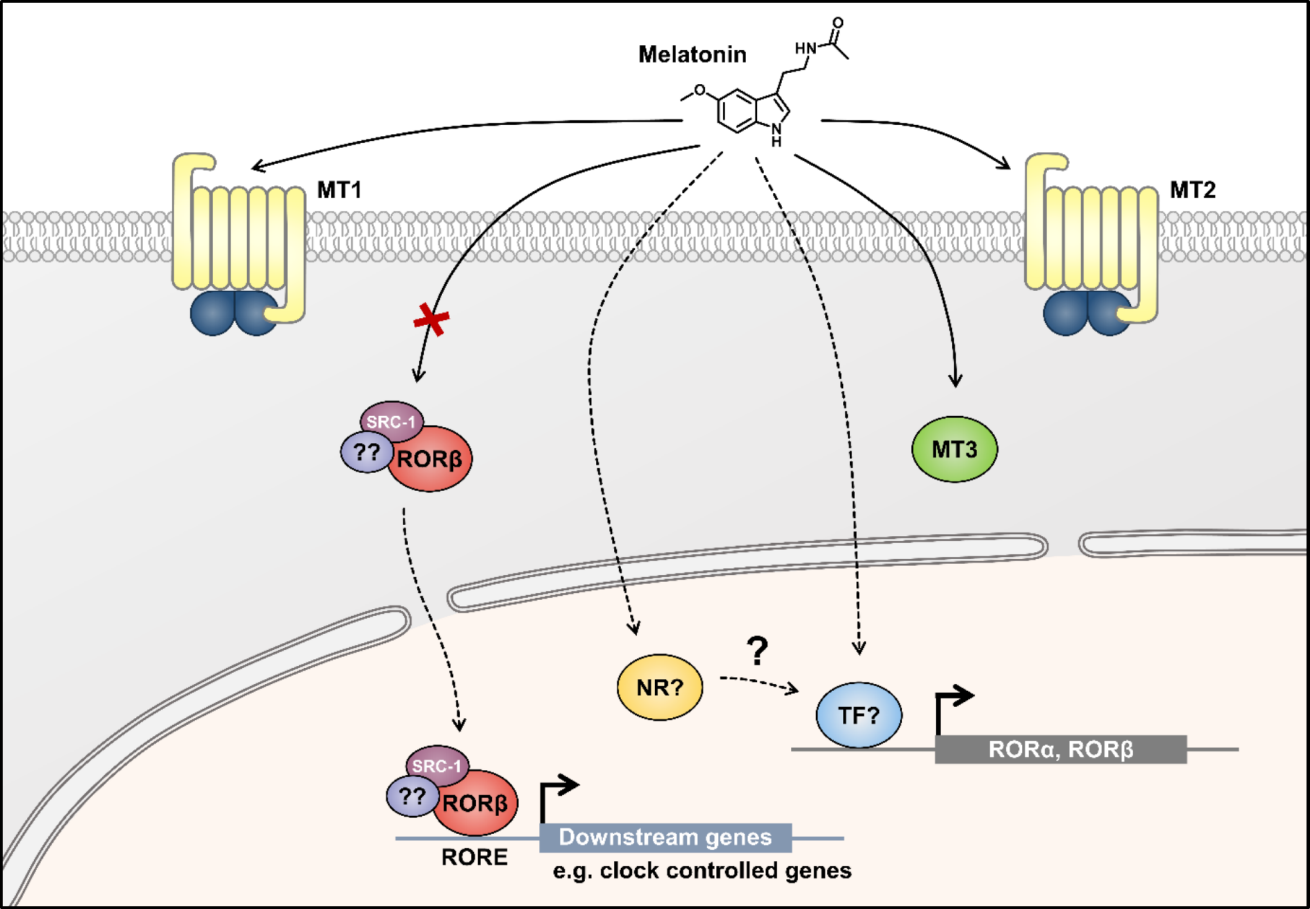
Melatonin was not the ligand for RORs. The actions of melatonin are mediated through signal transduction and its associated melatonin receptors, including the membrane-bound MT1/2 G protein-coupled receptors (GPCRs) and the cytoplasmic MT3 receptor, the latter having antioxidant and free radical scavenging characteristics. Herein, our results showed that melatonin was likely not the ligand for RORβ. Melatonin might bind to an unidentified nuclear receptor and influence the expression of RORs, which in turn affects the gene expression of the others

Taken together, our results indicated that melatonin was likely not the natural ligand for RORβ per se, while its effects on RORs might be mediated via its antioxidant properties or its downstream mediators, such as sirtuin 1 (Sirt1), regarding several physiological cellular roles of melatonin and RORs have in common [24, 37, 48]. In addition, melatonin may indirectly relate to RORs by regulating the expression levels of these genes. However, how melatonin is linked with the downstream effect of RORs remains the challenge, and the missing links are remained to be uncovered. Our postulation is that melatonin might bind to a yet-to-identify nuclear receptor and in turn regulates the expression of RORs, which affect the gene expression of the others (Fig. 8). We, therefore, urged the melatonin research community for the paradigm shift in the RORβ as the direct nuclear receptor for melatonin and warrant for the identification of nuclear target(s) of melatonin.

## Supplementary Information

Below is the link to the electronic supplementary material.

Supplementary file1 (DOCX 2.25 MB)

## Data Availability

All data supporting the findings of this study are available within the paper and its Supplementary Information.
