# Supplementary material for: A Complex Interplay Between Melatonin and RORβ: RORβ is Unlikely a Putative Receptor for Melatonin as Revealed by Biophysical Assays
Source: Mol Neurobiol. 2024 Aug 6;62(2):2333–47. doi: 10.1007/s12035-024-04395-y (PMC11772548; doi:10.1007/s12035-024-04395-y)
Supplement: Supplementary file 1 — Supplementary file1 (DOCX 2.25 MB) [file 12035_2024_4395_MOESM1_ESM.docx]

**Supplementary Information**

**Supplementary Table 1 Melatonin-binding proteins reported in the PDB database**

| **PDB entries** | **Proteins** | **Organisms** | **Resolution (Å)** | **Reference** |
| --- | --- | --- | --- | --- |
| 2QX6 | QR2 | *Homo sapiens* | 1.75 | Calamini et al, 2008 [49] |
| 2QX4 | QR2 | *Homo sapiens* | 1.65 | Calamini et al, 2008 [49] |
| 2QWX | QR2 | *Homo sapiens* | 1.50 | Calamini et al, 2008 [49] |
| 4QOG | QR2 | *Homo sapiens* | 1.40 | - |
| 4QOI | QR2 | *Homo sapiens* | 1.55 | - |
| 5I8F | Phenolic oxidative coupling protein | *Hypericum perforatum* | 1.30 | Sliwiak et al, 2016 [50] |
| 5MXW | Class 10 plant pathogenesis-related protein | *Lupinus luteus* | 1.57 | Sliwiak et al, 2016 [50] |
| 5MXB | Class 10 plant pathogenesis-related protein | *Lupinus luteus* | 1.51 | Sliwiak et al, 2016 [50] |
| 6TR5 | Palmitoleoyl-protein carboxylesterase Notum | *Homo sapiens* | 1.51 | Zhao and Jones, 2020 [51] |

**Supplementary Table 2 The LBD structures of RORβ in complex with ligands and co-activator proteins**

| **PDB entries** | **Organisms** | **ligands** | **Resolution (Å)** | **Reference** |
| --- | --- | --- | --- | --- |
| 1K4W | *Rattus norvegicus* | *Steric acid* | 1.9 | Stehlin et al, 2001 [16] |
| 1N4H | *Rattus norvegicus* | *Retinoic acid* | 2.10 | Stehlin et al, 2003 [17] |
| 1NQ7 | *Rattus norvegicus* | *7-(3,5-ditert-butylphenyl)-3-methylocta-2,4,6-trienoic acid* | 1.50 | Stehlin et al, 2003 [17] |

**References:**

16. Stehlin C, Wurtz JM, Steinmetz A, Greiner E, Schüle R, Moras D, Renaud JP (2001) X-ray structure of

the orphan nuclear receptor RORbeta ligand-binding domain in the active conformation. EMBO J

20(21):5822–5831. <https://doi.org/10.1093/emboj/20.21.5822>

17. Stehlin-Gaon C, Willmann D, Zeyer D, Sanglier S, Van Dorsselaer A, Renaud JP, Moras D, Schüle R

(2003) All-trans retinoic acid is a ligand for the orphan nuclear receptor ROR beta. Nat Struct Biol

10(10):820–825. https://doi.org/10.1038/nsb979

49. Calamini B, Santarsiero BD, Boutin JA, Mesecar AD (2008) Kinetic, thermodynamic and X-ray structural

insights into the interaction of melatonin and analogues with quinone reductase 2. Biochem J

413(1):81–91. <https://doi.org/10.1042/bj20071373>

50. Sliwiak J, Dauter Z, Jaskolski M (2016) Crystal structure of Hyp-1, a hypericum perforatum PR-10

Protein, in complex with melatonin. Front Plant Sci 7:668. <https://doi.org/10.3389/fpls.2016.00668>

51. Zhao Y, Ren J, Hillier J, Jones M, Lu W, Jones EY (2020) Structural characterization of melatonin as an

inhibitor of the Wnt deacylase Notum. J Pineal Res 68(2):e12630. https://doi.org/10.1111/jpi.12630

**
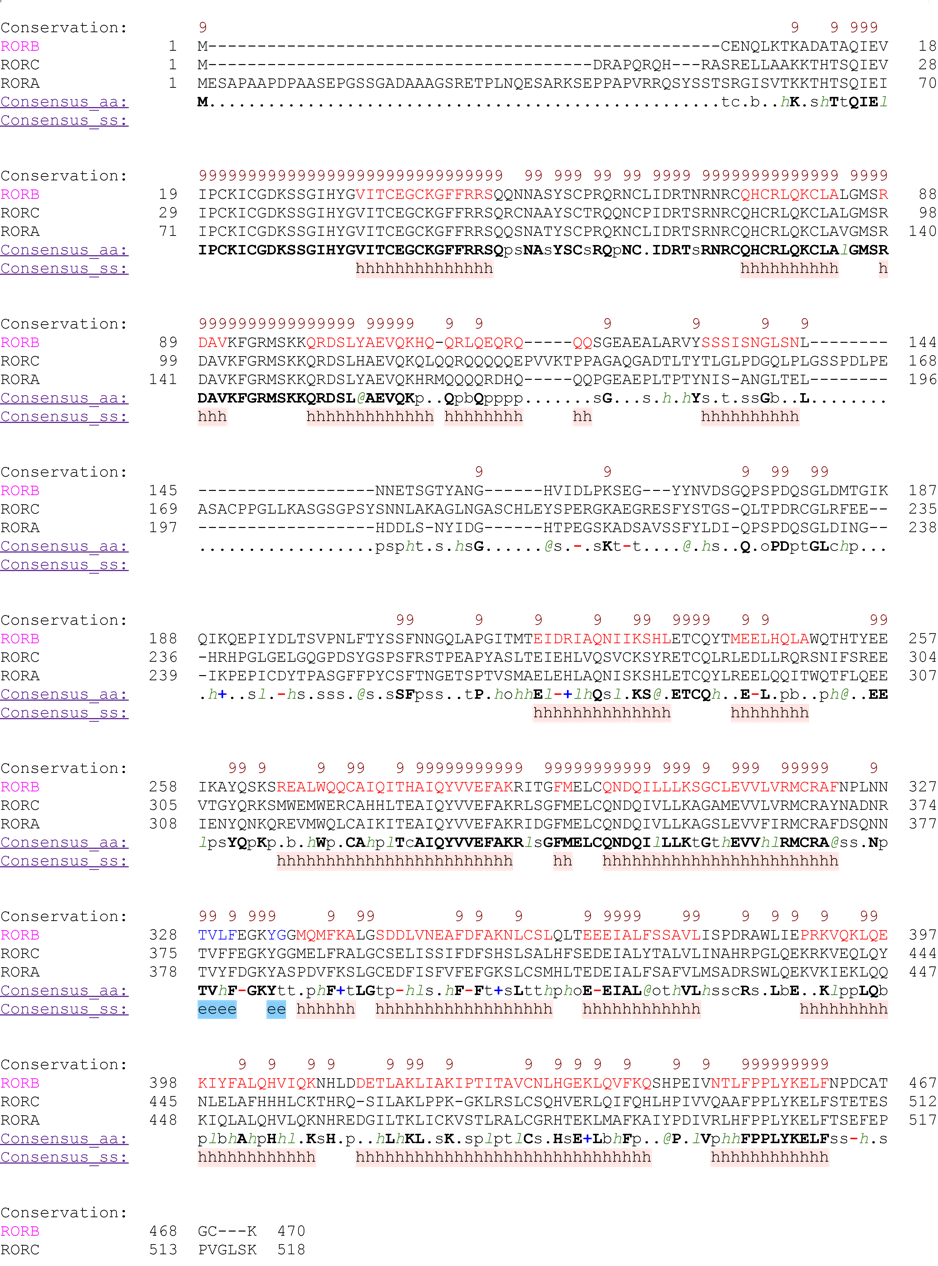
**

**Supplementary Figure 1 PROMALS3D alignment**

The first line in each block shows conservation indices for positions with a [conservation index](http://www.ncbi.nlm.nih.gov/entrez/query.fcgi?cmd=Retrieve&db=PubMed&list_uids=11524371&dopt=Citation). The values range from 4 to 9; high value indicates high level of conservation. The last two lines show consensus amino acid sequence (Consensus_aa) and consensus predicted secondary structures (Consensus_ss). Representative sequences have magenta names, and they are colored according to predicted secondary structures (red: alpha-helix, blue: beta-strand). Consensus predicted secondary structure symbols: alpha-helix: h; beta-strand: e. Consensus amino acid symbols are: conserved amino acids are in bold and uppercase letters; aliphatic (I, V, L): *l*; aromatic (Y, H, W, F): *@*; hydrophobic (W, F, Y, M, L, I, V, A, C, T, H): *h*; alcohol (S, T): o; polar residues (D, E, H, K, N, Q, R, S, T): p; tiny (A, G, C, S): t; small (A, G, C, S, V, N, D, T, P): s; bulky residues (E, F, I, K, L, M, Q, R, W, Y): b; positively charged (K, R, H): **+**; negatively charged (D, E): **-**; charged (D, E, K, R, H): c.

**
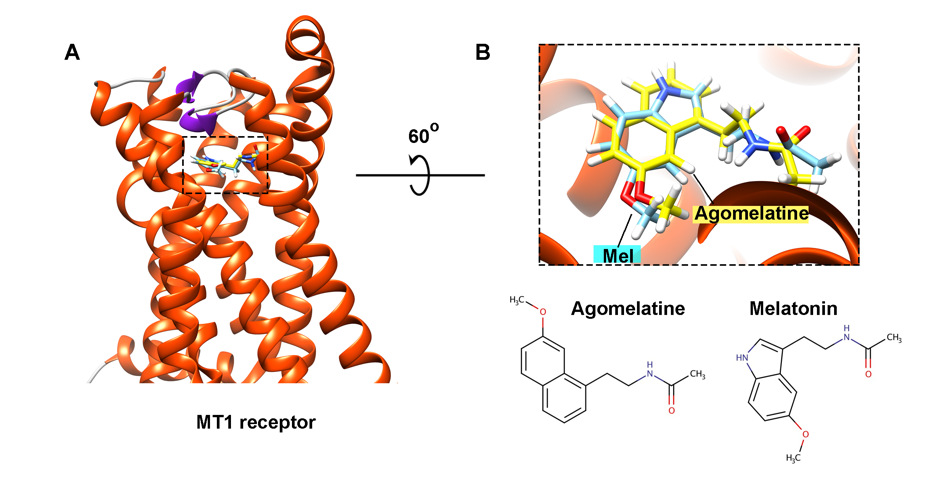
**

**Supplementary Figure 2** **Prediction of melatonin binding sites on the MT1 receptor**

**(A)** The MT1 receptor (PDB: 6ME5) was used to perform molecular docking of its natural ligand, melatonin. The melatonin-MT1 complex from docking prediction and the agomelatine-MT1 interaction site solved by X-ray crystallography are illustrated. The receptor is represented by ribbons (α-helices, vermilion; β-sheets, purple; connecting loop, grey). **(B)** The ligands in melatonin-MT1 and agomelatine-MT1 complexes are nearly superimposed. Ligands are depicted as sticks with oxygen, hydrogen, and nitrogen atoms colored in red, white, and blue, respectively. The molecular structures of melatonin and agomelatine are illustrated.
